# Potential Uses of Spent Coffee Grounds in the Food Industry

**DOI:** 10.3390/foods11142064

**Published:** 2022-07-12

**Authors:** Adriana S. Franca, Leandro S. Oliveira

**Affiliations:** Mechanical Engineering Department—DEMEC, Universidade Federal de Minas Gerais, Av. Antônio Carlos 6627, Belo Horizonte 31270-901, MG, Brazil; leandro@demec.ufmg.br

**Keywords:** polysaccharides, phenolics, antioxidants, polymer composites, bioplastics

## Abstract

Current estimates place the amount of spent coffee grounds annually generated worldwide in the 6 million ton figure, with the sources of spent coffee grounds being classified as domestic (i.e., household), commercial (i.e., coffee houses, cafeterias and restaurants), and industrial (i.e., soluble and instant coffee industries). The majority of the produced spent coffee grounds are currently being inappropriately destined for landfills or to a form of energy recovery (e.g., incineration) as a refuse-derived fuel. The disposal of spent coffee in landfills allows for its anaerobic degradation with consequent generation and emission of aggressive greenhouse gases such as methane and CO_2_, and energy recovery processes must be considered an end-of-life stage in the lifecycle of spent coffee grounds, as a way of delaying CO_2_ emissions and of avoiding emissions of toxic organic volatile compounds generated during combustion of this type of waste. Aside from these environmental issues, an aspect that should be considered is the inappropriate disposal of a product (SCG) that presents unique thermo-mechanical properties and textural characteristics and that is rich in a diversity of classes of compounds, such as polysaccharides, proteins, phenolics, lipids and alkaloids, which could be recovered and used in a diversity of applications, including food-related ones. Therefore, researchers worldwide are invested in studying a variety of possible applications for spent coffee grounds and products thereof, including (but not limited to) biofuels, catalysts, cosmetics, composite materials, feed and food ingredients. Hence, the aim of this essay was to present a comprehensive review of the recent literature on the proposals for utilization of spent coffee grounds in food-related applications, with focus on chemical composition of spent coffee, recovery of bioactive compounds, use as food ingredients and as components in the manufacture of composite materials that can be used in food applications, such as packaging.

## 1. Introduction

**Figure 1:**
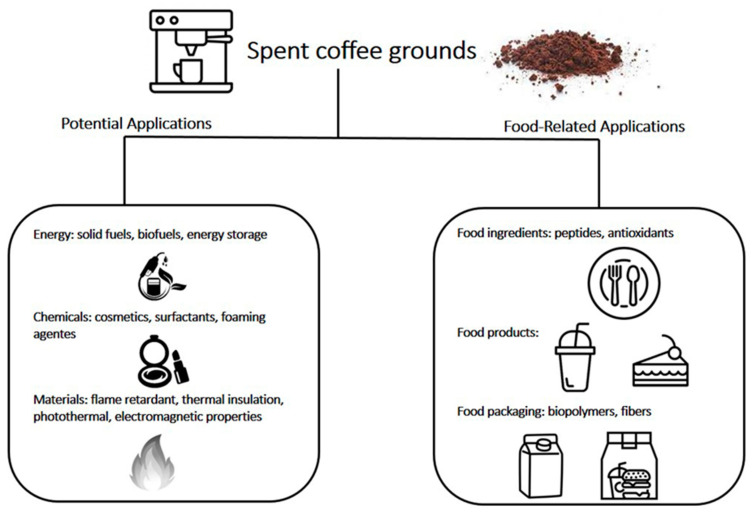
Summarized illustrative overview of potential uses for spent coffee grounds.

Spent coffee grounds (SCG) are roasted and ground coffee beans that were depleted of some of their water-soluble compounds. They are the solid residues obtained after coffee beverage preparation and can be found in a variety of places including homes and commercial establishments that serve coffee [1]. Disposal of SCG is quite a problem from an environmental point of view. These residues are usually thrown directly in the trash, and thus mostly end up in landfill sites, being highly pollutant due to significant amounts of organic substances that demand great quantities of oxygen to decompose [1]. Aside from environmental implications, SCG presents an additional disposal problem, because they can be used for adulteration of roasted and ground coffee and are very difficult to detect [2,3]. Thus, the soluble coffee industry must be extra careful with its disposal, and most of the time this residue is simply used as a boiler fuel by the same industry. Many recent studies have focused on finding alternative uses for SCG, and some applications are compiled in Table 1 and illustrated in Figure 1.

As is quite common for agricultural wastes in general [4], the first attempts towards finding alternative uses for SCG were concentrated on applications as solid fuels, fertilizers and as supplements for animal feed [1]. Among these applications, recent studies have proposed its potential use in a sustainable biorefinery route [5]. Such an approach focuses on the integrated use of various processes such as extraction, transesterification, hydrolysis, fermentation, and pyrolysis, resulting in several fuel types, including not only solid fuel pellets, but also biogas, bio-oil, biodiesel, and bioethanol. Applications in composting and soil amendment have been proven successful [6,7,8], and SCG were shown to favor the assimilation of nutrients by plants, even though it should be used in small quantities. Although a recent study has indicated that SCG is not suitable for vermicomposting [9], another research indicated that spent coffee grounds can be viewed as a source of biochelates to obtain fortified foods [10]. The chelating treatment increased SCG Fe content 108-fold and Zn content 18,000-fold. Functionalized SCG was able to significantly increase both Fe and Zn levels in lettuces. Fe levels increased by approximately 30%, whereas Zn levels showed a more significant increase, up to 400%.

SCG has also been suggested as an alternative feed source for livestock. Earlier studies have focused on the use of SCG as a feed ingredient in ruminant rations. Early in vitro studies with livestock observed that dry matter digestibility decreased as the amount of SCG increased [11], and this observation still holds for later studies [12]. In these studies, SCG was used to replace feed ingredients that were nutritionally richer, thus reducing the energy and nutrient content of the ration. Nonetheless, a recent study evaluated the potential use of SCG at lower doses as a functional ingredient in the diet of dairy sheep [13]. Increasing doses of SCG up to 100 g/kg (feed dry matter) resulted in increases in milk yield, protein and fat content. Additionally, no differences were found in intake, apparent dry matter digestibility and feeding behavior, indicating that inclusion of SCG provided improvements in milk production and composition without impairing feeding behavior or apparent digestibility.

**Table 1:** Recent proposals for alternative uses of spent coffee grounds.

Starting Material	Technology	Product/Application	Ref.
		**Energy**	
wood sawdust and SCG	pellet pressing	solid fuel pellets	[14]
SCG and larch sawdust or spruce shavings	high-pressure hydraulic briquetting press	solid fuel briquettes	[15]
SCG	pyrolysis	biochar/carbon cloth electrode/electricity generation and storage	[16]
SCG and reduced graphene oxide	vacuum-assisted impregnation	composite phase change material/solar energy storage	[17]
SCG	drying + oil extraction + transesterification	biodiesel	[18]
SCG	carbonization + CO_2_ activation	energy storage	[19]
		**Chemicals**	
SCG extract and *Xanthophyllomyces dendrorhous*	drying, sterilization and water extraction	astaxanthin/cosmetics, supplements and food	[20]
SCG extract and *Millerozyma farinosa*	drying, sterilization and water extraction	glutathione/medicine, food supplements and cosmetics	[21]
SCG	defatting + drying + hot water extraction + incubation with ammonium sulfate + centrifugation + ultra-filtration + vacuum drying	surfactants	[22]
SCG	solvent extraction (ultrasound, microwave or β-cyclodextrin-assisted)	phenolics	[23]
SCG	high-pressure temperature extraction	chlorogenic acids and caffein	[24]
SCG	subsequent extractions (hexane, water and ammonium sulfate) + centrifugation+ ultrafiltration + vacuum drying	foaming agent	[25]
		**Adsorbents**	
SCG	mixture with KOH and carbonization	biochar/ammonia removal from water	[26]
SCG	torrefaction	biochar/removal of diesel mixed in water	[27]
SCG	activation with NaOH, CaCO_3_ and carbonization	activated carbon/removal of methylene blue and methyl orange from water	[28]
SCG	no treatment	biosorbent/heavy metal (Cd) removal from aqueous solution	[29]
SCG	H_3_PO_4_ pyrolysis	microporous AC/removal of explosives from water	[30]
SCG	pyrolysis at 500 °C	biochar/removal of Norfloxacin (antibiotic) from water	[31]
SCG	washing with NaOH following by drying	biosorbent/recovery of dissolved metals (Fe, Al, Ca, Co, Mn, Ni, and Zn) from acid sulfate soil drainage	[32]
		**Materials**	
SCG and epoxy resin	mixing and curing	Flame retardant in polymer	[33]
SCG and Polylactide (PLA) powder	decolorization, micro- and nano-processing and extrusion	polylactic acid composite for 3D-printing	[34]
SCG and pectin	continuous casting	biocomposite pectin film	[35]
SCG and plaster	mixture with water and drying	thermal insulation material	[36]
SCG and hydrogels	near infra-red laser irradiation and incorporation into poly(N-isopropylacrylamide) hydrogels	photothermal materials	[37]
SCG	carbonization + composite mixture (cyanate ester, graphene nanoplates and epoxy resin) +hot pressing	composite with electromagnetic interference (EMI) shielding properties	[38]
		**Food products and ingredients**	
SCG	acid extraction followed by precipitation	bioactive peptides with antihypertensive and antioxidant potentials	[39]
SCG	microwave-assisted extraction of antioxidants, fermentation (Saccharomyces cerevisiae) and distillation	fermented and distilled alcoholic beverages	[40]
SCG	hydrolysis followed by fermentation with wine yeasts	alcoholic beverages	[41]
SCG	ethanolic extraction followed by addition of caramel, water, glucose syrup and vanillin	coffee-flavored liquor	[42]
SCG	extraction with isopropanol	antimycotic and anti-ochratoxigenic material; potential food ingredient with moderate cytotoxic and antibacterial activities	[43]

Many applications of SCG that are of interest for the food industry are related to the fact that moderate daily consumption of coffee can be associated with positive health effects [44]. Recent studies have confirmed that both green and roasted coffee extracts present inverse correlation with several diseases such as depression, Alzheimer’s, cancer, Parkinson’s, and diabetes, among others [45,46,47,48,49,50]. Coffee also shows protective effects on various systems including nervous, skeletal, reproductive, immune, and cardiovascular [51,52,53]. These health-related benefits are due to coffee’s rich phytochemistry, which includes substances such as caffeine and other biologically active compounds predominantly belonging to the polyphenol and alkaloid classes [1,54]. These substances are present not only in coffee beans or the associated beverage, but also in the solid wastes generated during coffee preparation, e.g., spent coffee grounds. Thus, in the following section, the chemical composition of SCG will be addressed to be later associated with potential food-related applications: the recovery of bioactive substances and development of food products and polymers that can be used in food packaging.

## 2. Chemical Composition

A comparative overview of the chemical composition of spent coffee grounds in comparison to green and roasted coffee is summarized in Table 2. The SCG proximate composition is expected to be somewhat similar to that of roasted coffee beans because SCG represents the solid matrix that remains after water extraction of soluble roasted coffee components. Protein contents are in the range of 12 to 15 g/100 g for roasted coffees, and of 10 to 17 g for SCG [55,56], so there are no significant differences in terms of protein contents. This class of compounds remains relatively stable during roasting and is not significantly extracted during beverage preparation. Nonetheless, reported contents are based on determination of total nitrogen, being probably overestimated due to the presence of other nitrogen-containing substances (e.g., caffeine, melanoidins, Maillard reaction products). Mineral contents range from 4 to 5 g/100 g in roasted coffee and are much lower in SCG (0.1 to 1 g/100 g), due to their significant extraction during beverage preparation. Lipid contents are reported to be in the range of 15 to 20 g/100 g and of 11 to 16 g/100 g for Arabica and Robusta roasted coffees, respectively. In the case of spent coffee grounds, lipid contents are reported to be in the same range of 22 to 27 g/100 g [1]. Given that lipids are relatively preserved intact during roasting (i.e., minor losses) and are not significantly extracted during beverage preparation, the higher values observed for SCG in comparison to roasted coffees are attributed to the differences in the dry mass basis used for calculations. Results for roasted coffee are expressed on dry basis of roasted coffee, whereas SCG results are expressed on dry basis of spent coffee grounds, with the dry mass of the latter being smaller due to the loss of soluble substances during extraction of the roasted and ground beans. The fact that such an effect was not present in protein contents is attributed to the extraction of nitrogen-based substances during beverage preparation. The fatty acid composition of SCG oil is reported to be similar to that of roasted coffee, with linoleic and palmitic acids being the predominant ones, respectively, at 43–50% and 32–43% [57]. The sterol content of SCG oils was determined to be in the range of ~3.5 to 11.5 g/100 g, and both the content and composition of SCG oil varied depending on the method of extraction [57,58]. However, regardless of the extraction method, the predominant sterols were sitosterol (~5 g/100 g) and stigmasterol (~4 g/100 g) [59,60]. Considering the significant contents of sterols, Nzekoue et al. [58] deemed SCG oils to be a potential alternative source of such compounds in regard to commercially produced sterols to satisfy their increasing demand in the global market [58]. Caffeine (1,3,7-trimethyl xanthine) is probably the most frequently ingested pharmacologically active substance in the world, mainly due to the popularity of coffee and other caffeinated beverages, and it is also used as adjuvant analgesic in combination with drugs such as acetaminophen, aspirin and ibuprofen [61]. Caffeine contents range from approximately 1 to 2 mg/g in roasted coffee, but the concentration in SCG is much lower because of the extraction that occurs during beverage preparation. Caffeine levels in SCG vary considerably, ranging from 0.7 to over 40 μg/mg [62]. Such variations are not only related to species (Robusta contains twice the caffeine content of Arabica), but also to extraction conditions and the type of solvent employed. High caffeine values are usually obtained with solvents that are commonly employed for decaffeination (dichloromethane, water and CO_2_), thus being already established as good extractants for this compound [62,63]. Naturally, the lowest caffeine contents of SCG are observed for coffees that underwent decaffeination.

Coffee beans are a rich source of polysaccharides, mostly cellulose, galactomannans and arabinogalactans [64]. Except for cellulose, roasting increases the polysaccharides’ solubilities because the cell wall structure loosens as the coffee beans swell and the degrees of polymerization and of branching of the hemicelluloses are significantly altered. Hence, a considerable portion of the hemicellulose polysaccharides will be removed by water extraction during coffee beverage preparation. Oligomers and monomers are rapidly converted into Maillard and pyrolysis products during roasting. Arabinoses present as side-chains in the arabinogalactan molecules are deemed more susceptible to thermal degradation than galactoses and mannoses as roasting conditions become more severe [65]. Regardless of this increase in solubility, a substantial fraction of these polysaccharides are retained as insoluble material bound to the SCG matrix [66,67]. Galactomannans were determined to comprise 50% of the polysaccharide fraction of SCGs, whereas cellulose and arabinogalactans account for the other 50% fraction in equal proportions [67,68,69]. Average monosaccharide compositions of SCG were reported in the ranges of 37 to 46% for mannose, 27 to 32% for galactose, 20 to 24% for glucose, and 7% for arabinose [56].

As previously mentioned, SCG are rich in polysaccharides, mostly cellulose and hemicellulose. The polysaccharide fraction comprises neutral detergent fibers (45.2%) and acid detergent fiber (29.8%) [70]. The total dietary fiber (TDF) content of SCG ranges from 45 to 51 g/100 g, of which 35 to 48 g/100 g are insoluble fibers and 2 to 8 g/100 g are soluble fibers [71,72]. TDF contents of SCG are in the same range of fiber-rich powders from fruit peels such as orange (~49–51 g/100 g), prickly pear (~41 g/100 g) and feijoa (~46–48 g/100 g) [73,74] but higher than some commonly used fiber sources such as rice and wheat bran (~27–45 g/100 g) [75] and some fruit peel powders including pequi (40–43 g/100 g) and mango (40–41 g/100 g) [73,76]. Spent coffee grounds have lower TDF contents in comparison to other coffee processing residues, including coffee silverskin (~62 g/100 g) [77] and coffee husks and pulp (~66 g/100 g) [78], and also buriti peels (~89 g/100 g) [79]. However, Vilela et al. [71] demonstrated that, when treated with alkaline hydrogen peroxide, total dietary fiber content of SCG increased to 70 g/100 g.

Aside from differences in technological functionalities, the insoluble (IDF) and soluble (SDF) natures of dietary fibers will also present distinct physiological activities in the human body, with IDF being associated with the ability to increase fecal bulk and decrease intestinal transit and SDF associated with increased solution viscosity and reduced glycemic response and plasma cholesterol, as well as prebiotic action [75]. IDF fibers in SCG are in the range of 80 to 90% of the total dietary fiber. IDF contents of SCG are in the same range as those of lemon (~42 g/100 g), orange (~52 g/100 g) and carrot (~34–51 g/100 g) peels and of pomegranate bagasse (~29–30 g/100 g). Insoluble dietary fiber contents of SCG are significantly higher than the contents in apple, orange, dates and tomatoes (~5–12 g/100 g), but much lower than in buriti peels (~88 g/100 g) [75,79,80]. On the other hand, SDF contents of spent coffee grounds are lower than SDF contents of agricultural residues such as carrot peel and pomegranate bagasse (~9.8–19.9 g/100 g) [75,80]. SCG dietary fibers were categorized as antioxidant dietary fibers by Murthy and Naidu [72], since they exhibited antioxidant activities similar to those of red wine products, thus, being considered a potential dietary supplement.

The antioxidant activity of food products is usually associated with the contents and types of phenolic compounds in their composition. Phenolic compounds in coffees mostly comprise chlorogenic acids (CGA), corresponding to a class of water-soluble esters in which one or two moieties of caffeic or ferulic acids are esterified to a quinic acid molecule, with the most abundant CGA being caffeoylquinic, dicaffeoylquinic, feruloylquinic, and p-coumaroylquinic acids. The antioxidant activity and nutraceutical potential of green coffees were attributed to the CGA class [81,82,83]. Although CGA contents were repeatedly demonstrated to significantly decrease during roasting [84], comparative evaluations demonstrated that aqueous solutions from green and roasted coffees presented similar antioxidant activities. A slight decrease in CGA content was observed for light roasts, followed by an increase in darker roasts, with this behavior being respectively attributed to loss of phenolic compounds during light roasting and to the successive formation of Maillard reaction or pyrolysis antioxidant products in dark roasts. Therefore, the antioxidant potential of spent coffee grounds is probably due to the combined effect of the remaining CGA as well as Maillard reaction products that were not extracted during beverage preparation.

## 3. Applications

Recent reports and reviews on the use of SCG and other coffee processing by-products are available [85], including applications in the fabrication of construction materials [86], cosmetics [87], recovery of bioactive substances [88], biopolymers and biocatalysts [89,90], food packaging [91], materials and energy [92], as well as multiple uses considering a biorefinery approach [5,93,94,95,96,97,98,99]. In the next sections, we will comment on the latest findings on applications of SCG that are of particular interest for the food industry.

### 3.1. SCG as a Source of Bioactive Compounds

As mentioned in the previous section, SCG are rich in polysaccharides, polymers that can be used as dietary fibers, and present immunostimulatory activity [62]. Therefore, a few recent studies on the extraction of polymeric sugars from SCG are available in the literature. Bhaturiwala [100] proposed a series of sequential treatments for extraction of both oligosaccharides and phenolics from SCG. The treatments consisted of roasting at 150 °C for 30 min, followed by heating with water at 90 °C and then hydrolysis of the pretreated materials (both the solid and liquid residues) with β-mannanase. The enzymatic hydrolysis was performed at 45 °C for 20 h, with the solutions later heated for 10 min at 90 °C for enzyme deactivation. Both the materials obtained from the solid and liquid residues were then lyophilized for analysis. The total amount of reducing sugars increased to 106.10 (mg glucose/g) in the combined treated materials from an initial content of 5.32 (mg glucose/g raw SCG, while the total phenolic content (estimated by Folin–Cilcateau) increased from 45.68 mg gallic acid/g in the untreated SCG up to 291.86 (mg gallic acid/g lyophilized material). FTIR analysis indicated the breakage of the β-glycosidic bonds between cellulose and hemicellulose, confirming the efficiency of the extraction procedures. Zhang et al. [101] evaluated the extraction of polyphenols and polysaccharides from SCG using water with ultrasound assistance. The authors employed central composite design to maximize the yields of both polysaccharides and polyphenols. Polyphenol and polysaccharide yields of 1.0 and 1.1% wt. were obtained under optimal conditions (1:23 solid/water ratio, 213 W ultrasound power, 30 min extraction and 68 °C extraction temperature). Wongsiridetchai and collaborators [102] characterized the mannooligosaccharides obtained from SCG and evaluated their prebiotic properties. SCGs were submitted to an alkali treatment with NaOH and then digested by mannanase from *Bacillus subtilis* GA2. Characterization results showed that the alkali treated SCG mainly comprised mannose, mannobiose, mannotriose, and mannopentose. The results showed that the obtained mixture of manno-oligosaccharides had prebiotic properties (promotion of lactic acid bacteria growth, tolerance under gastrointestinal conditions, and pathogen growth inhibition), confirming the potential of SCGs as a prebiotic agent.

The recovery of phenolic compounds from SCG has been extensively investigated, given the considerable attention that this class of compounds has been receiving due to their beneficial effects on human health [62]. Different extraction techniques have been employed, with the earlier studies focusing on traditional solid–liquid extraction [72,103] and lately employing other techniques such as ultrasound [104,105], subcritical water extraction [106], pressurized liquid extraction [24,107], supramolecular solvent extraction [108] and microwave-assisted extraction [109], among others, in order to improve extraction conditions.

A simple solid–liquid extraction procedure was proposed by Ramón-Gonçalves and collaborators [110] for extracting polyphenols from spent coffee grounds. Extraction conditions were optimized based on chemometric analysis, and the best conditions obtained in this study were 15 min extraction at 60 °C using a 25:75 (*v*/*v*) ethanol–water solution. Chlorogenic acid, p-coumaric acid, trans-ferulic acid, rutin, naringin and resveratrol were extracted, with variations in quantities depending on the original coffee variety. Chlorogenic and p-coumaric acids were the polyphenols found in higher amounts. Both the composition and bioactivity of ethanolic extracts obtained from spent espresso coffee grounds were investigated in a recent study [111]. SCG samples were based on pure and mixed Arabica and Robusta coffees from distinct provenances but using the same brewing parameters. The following bioactive compounds were found: α- and β-tocopherols, chlorogenic acids, 4-hydroxybenzoic acid, vanillin and tyrosol. Among the evaluated samples, the extract obtained from 100% Robusta Guatemala coffee showed the highest α- to β-tocopherol ratio of antioxidant potential, and promising anti-proliferative activity toward human lung carcinoma cells. However, the significant variability in terms of chemical composition and bioactivities found in the evaluated samples should be considered in further studies. Arauzo and collaborators [112] extracted phenolic compounds from spent coffee grounds by a hydrothermal delignification method followed by ultrasound-assisted extraction. The hydrothermal treatment was carried out with three distinct solvents: water, methanol and a water–methanol mixture. The highest extraction of total phenolic content (11.7–20.3 mg GAE/mg of dry sample) was achieved with the ultrasound-assisted extraction applied to the hydrochars using methanol as a solvent, with the mildest delignification process conditions providing the highest extraction of phenols from the hydrochars. The hydrochars were considered promising solid biofuels, with fuel ratios of 0.3 to 0.7, energy yields ranging from 83 to 95.2%, and HHV values in the range of 28.5 to 33.2 MJ/kg.

The study by Bitencourt and collaborators [113] employed supercritical extraction methods for recovery of phenolics from SCG. Extraction was performed at 333 K and 40 MPa, in one or two steps, using the following solvents: pure supercritical CO_2_; ethanol; and a supercritical mixture of CO_2_ with ethanol (90:10 *w*/*w*). The use of high pressure was important to increase the overall extraction yields. Extractions performed with ethanol as solvent presented the highest overall extraction yield and total phenolics content. Liquid–solid extraction assisted by sonication was employed by Zengin et al. [114] for obtaining SCG extracts using several solvents: H_2_O, MeOH, MeOH:H_2_O (50:50), and EtOH:H2O (70:30). The ethanolic extract was the richest in caffeine and presented the highest antioxidant activities. However, the hydroalcoholic and methanolic extracts were shown to be the most effective in terms of enzymatic inhibitory activity against acetylcholinesterase, butyrylcholinesterase, α-amylase, α-glucosidase, and tyrosinase, while the water extracts displayed lower activity. Such results confirm SCG as a potential source of bioactive metabolites with biological activity. Nonetheless, variations in solvent polarity led to differences in the biological activities of the products, confirming that extraction is a crucial step in the recovery of bioactive compounds from SCG [114].

Isopropanol extracts obtained from SCG were investigated in terms of their antimycotic and anti-ochratoxigenic potentials [43]. The substances encountered in higher amounts were caffeine and the following phenolics: (R)-(+)-rosmarinic, syringic, gallic, sinapic, salicylic, chlorogenic and caffeic acids. Apigenin-7-glucoside, naringin, epicatechin, and catechin were the predominant flavonoids. The obtained extracts showed degradation efficiency against the growth of Aspergillus strains. The SCG extracts provided detoxification in liquid media for aflatoxins (AFs) and ochratoxin A (OCA) and antifungal effect against toxigenic fungal strains, especially *A. flavus* and *A. ochraceus*. Both ethanolic and aqueous extracts were also evaluated with respect to their antibacterial, antiradical and antiproliferative potentials [115]. Although slightly higher yields were obtained with water extraction, the amount of extracted phenolics was significantly higher with ethanol. In antiproliferative assays, SCG extracts exhibited a significant decrease in viability of both lung and cervical carcinoma (C33A) cell cultures, increased apoptotic cells and promoted cell cycle arrest. The higher total phenols content and antiradical activity of SCG ethanolic extracts were thus related to their antiproliferative activity in cancer cells and their antibacterial activity against clinical isolates [115].

A recent study [116] proposed the use of a tertiary amine (N,N-dimethylcyclohexylamine, CyNMe2) to simultaneously separate SCG into three fractions: a carbohydrate fraction; a phenolic fraction; and a lipid fraction consisting mostly of palmitic and linoleic acid. Although the yields of each fraction were influenced by extraction conditions, it was observed that 24 h extractions within a temperature range of 35–25 °C led to significant yields of carbohydrates (68–83%), phenolics (479–535 mg g^−1^), and lipids (9.97–11.54%). This study is of particular relevance in terms of technological features, given that the employed solvent was able to produce satisfactory yields of each fraction at once.

Although most of the works on the extraction of bioactive substances from SCG have focused on the extraction of polysaccharides, polyphenols and caffeine, a few recent studies have looked at the possibility of using SCG as a source of protein and peptides [117,118]. Valdés and collaborators [117] performed a comparative evaluation of two methods for protein extraction from SCG. It was observed that a urea-based extraction buffer allowed for a more efficient extraction of proteins in comparison to the use of Tris-HCl buffers. The highest protein content was obtained from espresso SCG. Furthermore, the produced protein hydrolysates were shown to have antioxidant and ACE-inhibitory activities. The study developed by Ramirez et al. [118] focused on obtaining protein hydrolysates with potential bioactivity after fermentation of SCG using *Bacillus clausii*. Fermentation was able to increase the amounts of total proteins, soluble proteins, and protein hydrolysates by 2.7, 2.2, and 1.2-fold, respectively. It is worth noting that there was an increase in the amounts of seven peptides that displayed potential antioxidant capacity, angiotensin-converting enzyme activity, and dipeptidyl peptidase-IV-inhibitor activity.

### 3.2. SCG in Food Products

As well established in the previous sections, extracts obtained from SCG can be a rich source of antioxidants, so they could be used as alternatives to synthetic antioxidants in many food-related applications, including inhibition of lipid oxidation, and thus ethanol (with and without heating) and hot water extracts were evaluated for that purpose [119]. It was observed that both ethanol and water SCG extracts presented high antioxidant activity and DPPH radical scavenging capacity. Among the evaluated extraction methods, heated ethanol extraction (80 °C for 1 h) was the most effective for extraction of antioxidants and in preventing lipid oxidation in oil emulsion and raw meat systems. However, the method was not able to prevent oxidative changes in cooked meat packaged in oxygen permeable bags for more than 3 days. Nonetheless, the potential of SCG extracts in avoiding lipid oxidation in meat products was established, and results could probably be improved by introducing modifications in the extraction methods and/or the antioxidant delivery method.

A few studies have dealt with the use of SCG in the production of beverages. Sampaio et al. [120] employed a three-step process to produce a spirit from SCG. The steps consisted of: (i) hydrothermal treatment, (ii) fermentation of the obtained extract supplemented with sucrose to ethanol, and (iii) distillation. Coffee was the most representative aroma based on aroma analysis, and sensory evaluation results indicated that the produced beverage presented pleasant flavor, with both coffee taste and smell. A process developed by Machado [40] was based on microwave-assisted water extraction of SCG, followed by fermentation of the obtained extract with *Saccharomyces cerevisiae*, after being supplemented with sucrose, and then distillation of the fermented broth. Two fermented and two distilled beverages were obtained, with ethanol contents varying from approximately 10 to 40% for the fermented and distilled beverages, respectively. The substances that affected both the aroma and flavor of the produced beverages were characterized by GC-MS, with almost 60 volatile components being identified. The fermented beverages were characterized by higher alcohols, including isobutanol and isoamylic alcohol, and esters that had a positive contribution to the beverage aroma. Alcohols as major compounds and volatile acids and esters as minor compounds were most abundant in the distillate beverages, contributing to the fruity flavor and floral aroma of the drink [40]. Overall, the distillate beverages presented better sensory quality, although the authors concluded that both the fermented and distilled beverages obtained based on SCG extracts presented acceptable organoleptic qualities. A recent study evaluated the potential of producing fermented beverages from SCG extract employing non-Saccharomyces wine yeasts, namely *Torulaspora delbrueckii* and *Pichia kluyveri* [41]. An SCG hydrolysate was prepared based on a sequential acidic/enzymatic hydrolysis method, pasteurized, and later fermented. It was observed that adding the yeast extracts improved the generation of succinic and lactic acids and the production of odor-active compounds such as short-chain esters. Although the study indicated the potential of developing novel fermented SCG-based products, no sensory data were available, so at this point there is no knowledge on the organoleptic aspects of the produced beverages. Masino and collaborators [42] employed extracts prepared by mixing SCG with absolute ethanol to prepare coffee-flavored liquor mixtures with water, caramel, and glucose syrup. It was concluded that the prepared hydroalcoholic solutions could be used as a substitute for coffee extracts or flavorings in the formulation of coffee-flavored liquor.

The fact that SCG can be viewed as rich sources of fibers has prompted some studies on its use in bakery products. Martinez-Saez et al. [121] evaluated the use of SCG as a food ingredient in biscuits. The results confirmed SCG as natural sources of antioxidant insoluble fibers and essential amino acids. SCG were added to biscuit formulations together with low-calorie sweeteners and oligofructose. Results indicated that SCG could be used directly as food ingredient in biscuits (up to 4% *w*/*w*) without affecting the final nutritional or sensory quality of the product. SCG and the residue obtained after supercritical extraction of its oil content (SCGR) were used for the preparation of cookies [122]. Both SCG and SCGR were compared in terms of textural properties, bioactivity, prebiotic activity, and sensory analysis. No significant differences were observed in the probiotic viability count of both types of cookies, indicating that the polysaccharide profile did not change significantly with the oil extraction. Results from the sensory analysis indicated reasonable acceptability (score 7 out of 10) for cookies with up to 7% (*w*/*w*) SCGR or SCG in comparison to the control samples with overall acceptability of 9. Use of larger concentrations of SCGR increased the bitterness of the cookies and adversely affected both the texture and overall acceptability. Nonetheless, this study confirmed the potential of SCG as bakery ingredients. In addition, the extracted SCG oil was shown to be rich in polyphenols and presented a mild bactericidal effect, and thus could also be used as a functional ingredient in other food products.

SCG was also evaluated as a functional ingredient in sponge cakes [123]. It was observed that flour substitution with SCG (2, 4 and 6%) led to a reduction in the degree of browning, this being attributed to the lower glycemic sugar protein contents in comparison to the control sample. SCG supplementation resulted in production of sponge cakes with significantly higher volume, weight, and specific volume in comparison to the control sample. Texture profile analysis data indicated that adding SCG to the cake formulation resulted in decreases in hardness, resilience, cohesiveness, springiness, gumminess, and chewiness of the product. Addition of SCG had a deleterious effect on the organoleptic properties, although cakes prepared with 2 and 4% SCGs presented significantly higher sensory scores in comparison to cakes supplemented with 6% SCGs. The oil extracted from spent coffee grounds was used by Meerasri and Sothornvit [124] as a partial replacement for butter (10 to 30%) in cookies, with the substitution content of 20% being the limit at which cookie textural and organoleptic properties were considered acceptable by trained panelists. The partial replacement of butter by SCG oil increased both the phenolic contents and the antioxidant properties and softened the texture of the cookies.

### 3.3. SCG in Polymers

Polymeric materials have been extensively used as packaging materials given that they are lightweight in nature, relatively easy to produce, resistant to corrosion, and have appropriate mechanical and thermal properties, among other characteristics [125]. In the case of food products, packaging aims mainly to avoid food deterioration during several steps including production, storage, distribution, sales and even handling at home. Lately, the functions of food packaging have been extended to include increased shelf-life, waste minimization and marketing [126].

Some of the recent advances in terms of polymer-based food packaging research are associated with reducing their environmental impact by employing biodegradable polymers as alternative materials to conventional fossil fuel-based plastics. Since agricultural wastes such as SCG are a rich source of polysaccharides, a few studies have focused on the production of biopolymeric films using the polysaccharide-rich fraction of SCG as precursor material [127,128]. Galactomannans, which represent the main fraction of polysaccharides present in SCG, are high molecular weight molecules with low branching degree, comprising a backbone of (β1→4)-linked mannose residues and sporadic units of (β1→4)-linked glucose, with O-6 single (α1→6)-linked galactoses and single (α1→5)-linked arabinose residues. SCG galactomannans are insoluble in water and form highly viscous and stable aqueous solutions of ZnCl_2_ that have film-forming ability, thus being quite interesting raw materials for the production of edible and biodegradable films or coatings for food applications [91,127,128].

Biofilms that were predominantly composed of galactomannans were produced using SCG submitted to alkaline and enzymatic treatments [127,128]. The treatment with alkaline hydrogen peroxide was employed to remove lignin and phenolics, whereas a subsequent enzymatic treatment was used to partially remove cellulose. It was observed that interactions between the film matrix polysaccharides had a direct influence on the film properties. The authors reported that cellulose plays an important role in film formation as it influences crosslinking and, although tensile strength values were similar for films with and without enzymatic treatment, cellulose removal increased both Young’s modulus and elongation at break. Film failures during the mechanical tests were attributed to the presence of aggregates, probably of polysaccharides mediated by chlorogenic acids. Nonetheless, it was shown that the developed biofilms have extensive application potential not only in the food industry, but also in the engineering field in general.

Besides pure polymers, polymer composites have also been extensively investigated in recent years. In this context, SCG have been evaluated as fillers, aiming to improve the polymeric matrices’ mechanical, thermal, and barrier properties, and as additives that can improve specific properties such as antioxidant characteristics [125]. A compilation of recent studies that deal with the application of SCG in polymer composites is shown in Table 3. One of the major difficulties found in the addition of SCG as fillers is the poor compatibility between the filler and the polymeric matrix. Therefore, several studies employed chemical treatment of the SCG to modify the respective surface properties and improve SCG particle adhesion to distinct polymeric matrices, as shown in Table 2. Furthermore, SCG and other types of natural fibers are mainly composed of cellulose, hemicelluloses and lignin, and these polysaccharides have hydrophilic hydroxyl groups in their structures. These groups form new hydrogen bonds with water molecules present in the air, thus absorbing moisture. Pectin and waxy substances hold these water molecules, hindering free hydroxyl groups from bonding with the polar matrix. As a result, a poor interfacial bonding between the polysaccharides and matrix occurs.

Most of the studies dealing with SCG in polymer composites have used petroleum-based polymeric matrices. Nonetheless, to obtain a truly green composite comprising 100% renewable materials, not only the fiber but also the polymer should be bio-based [131]. With that in mind, a study by Tarazona et al. [131] demonstrated that it was possible to obtain a polymeric matrix by epoxidation of waste soybean oil and use SCG as a reinforcement to improve the composite mechanical properties. Other studies have prepared composites using pectin [35] and corn starch [135]. Although the employed polymers were of commercial grade, these can be easily recovered from food and agricultural wastes, so there is much potential in obtaining completely waste-based polymer composites using SCG and other agricultural residues.

A few studies have employed some forms of extract obtained from SCG as additives in polymer composites. In a recent study developed by da Silva et al. [138] coffee oil extracted from SCG was used as a plasticizer for development of a composite based on cellulose obtained from recycled paper coffee cups. The paper cups were milled, modified with lactic acid, and then mixed with poly(lactic acid) (PLA). The addition of coffee oil improved both the flexibility and processibility of the resulting polymer. The aqueous extract obtained from spent coffee grounds (after water extraction at 100 °C for 5 min) was employed together with citric acid as an additive in polyvinyl alcohol (PVA)/starch films [139]. The incorporation of SCG extract improved antioxidant release and antimicrobial activity, while showing a synergistic effect with citric acid on film antibacterial activity. Although there was a slight decrease in tensile strength with the addition of citric acid, the produced films were shown to be adequate for the development of new green antimicrobial materials for application in food packaging. SCG extracts were also used as additives in a poly(lactic acid) (PLA) polymer matrix using diatomaceous earth as reinforcing filler [140]. Improvements in both mechanical and oxygen barrier properties were observed for the polymers characterized by the co-presence of diatomite and SCG extract, indicating a possible synergistic effect of the two additives. Getashew et al. [141] developed packaging films using gelatin extracted from fish skin impregnated with SCG extracts obtained by subcritical water extraction. The films presented high antioxidant and antimicrobial activities against several food poisoning bacteria, including *Bacillus cereus*, *Staphylococcus aureus, Listeria monocytogenes* and *Escherichia coli*. These results confirmed the potential of using SCG and other types of agricultural and food wastes in the production of active food packaging.

Spent coffee grounds were incorporated at 20% wt. into PLA by extrusion to develop a green composite [136]. Two oligomers of lactic acid, one not functionalized and the other functionalized with maleic anhydride, were mixed with SCG during an extrusion process, resulting in composites with ductility increased by approximately 280%, which was attributed to the high lipid content of SCG. Increased tensile strength was also observed, with the functionalized PLA oligomer contributing to an increased strength of about 60% due to increased chemical interactions between the biopolyester and the SCG fillers promoted by the maleic anhydride functional groups. The resultant composites were deemed promising alternatives for the production of disposable food-serving utensils and tableware. Papadaki and co-workers [142] investigated the incorporation of an antioxidant-rich extract obtained from spent coffee grounds into whey protein edible films with the aim of improving the functional properties of the films. The incorporated SCG extract promoted an increase in both the tensile strength and the elastic modulus of the whey protein films. The inclusion of SCG phenolics into the protein films improved their UV-light resistance and increased the antioxidant properties of the films. The antioxidant capacity of films remained high after a 12-month storage period at 5 °C, decreasing only 16.8% in relation to its original activity. The supplemented films were thus considered promising active packaging material for long-term storage of foods with lipid oxidative susceptibility.

Recent comprehensive reviews are available in the scientific literature on the topic of composite materials produced with spent coffee grounds and other coffee by-products that could be used for food applications [91,92,143].

## 4. Conclusions and Future Perspectives

Spent coffee grounds recently have been extensively researched as precursor material for a variety of value-added applications, including biofuels, catalysts, cosmetics, composites, and food ingredients. Spent coffee grounds are rich in a diversity of classes of compounds, such as polysaccharides, proteins, phenolics, lipids and alkaloids, and as such, are suitable candidates for applications in food-related areas. An extensive body of work has been recently published on food-related applications of SCG with a focus on recovery of food-grade bioactive compounds (e.g., chlorogenic acids and its derivatives, and caffeine) that can be further used in food formulations, food ingredients (e.g., fiber), and precursors for composite materials for food packaging (e.g., biopolymeric films and composite fillers). The repurposing, recycling, and upcycling of SCG, avoiding or delaying its disposal in landfills or use as refuse-derived fuel, not only minimize environmentally negative impacts in the coffee production and consumption chain but also allow for an extended service lifecycle of coffee. Increased revenues would also be another advantage of extending the coffee service lifecycle if the soluble and instant coffee industries could themselves further process SCG into value-added food-related products, employing the concepts of biorefinery and circular economy. Most of the current research has been focused on developing a single product out of SCG, and a handful of published works have presented possible scenarios for application of biorefinery concepts to coffee by-products. However, with the development and use of innovative technologies (e.g., ultrasound, microwave and others) for extraction, fractionation and purification of compounds from spent coffee grounds, a wider spectrum of applications can be envisioned for SCG-derived products. Future investigations should consider detoxification and subsequent hydrolysis of SCG compounds, thus allowing the application of a diversity of biotechnological processes for the conversion of SCG into food-grade ingredients, alleviating the need to use actual agricultural and food products for such endeavors.

## Figures and Tables

**Table 2:** Comparative overview of the chemical composition of spent coffee grounds in comparison to green and roasted coffee (g/100 g dry basis).

	Green Arabica and Robusta	Roasted Arabica	Roasted Robusta	Spent Coffee Grounds
Protein	13–17	12–15	13–15	10–17
Lipids	9–15	15–20	11–16	22–27
Minerals		4–5	5	0.1–1
Carbohydrate		40–79	64–71	45–89
Caffeine		~1	~2	0.07–0.4
TDF	-			45–51
Cellulose	6.7–8.7	-	-	16–25
Galactomannans	25	12–13	-	~23
Arabinogalactans	17	-	-	~11
Protein	13–17	12–15	13–15	10–17
Lipids	9–15	15–20	11–16	22–27
Minerals		4–5	5	0.1–1

**Table 3:** Some recent applications of SCG as fillers in polymers.

SCG Treatment	Polymer/Treatment	Main Effects of SCG Addition	Ref.
Removal of coffee oil by hexane extraction by ultrasonication	Polypropylene/extrusion	Improvement in mechanical and thermal properties	[129]
Torrefaction	poly(butylene adipate-coterephthalate)/extrusion	Enhancement in the thermo-mechanical properties and increased hydrophobicity	[130]
Alkali treatment	Epoxidized soybean oil/heating and curing	Significant improvement in mechanical properties	[131]
Alkali treatment	Cellulose/casting	Increase in tensile strength and thermal stability	[132]
Alkali treatment, bleaching and mixture with coupling agent	Polypropylene/extrusion	Improvement in mechanical properties	[133]
none	Poly(3-hydroxybutyrate-*co*-3-hydroxyvalerate)/casting	Decrease in tensile strength, slight increase in elongation	[134]
none	Pectin/continuous casting	Increase in water permeability/improvement in thermal properties	[35]
none	Starch/microwave heating	Increase in tensile strength/no effect on thermal properties	[135]
Milling (SCG from ethanolic extraction)	Polylactide + lactic acid oligomers/extrusion	Increase in ductility	[136]
Slow pyrolysis	Polyethylene terephthalate + linear low-density polyethylene/extrusion	improvements in the flexural modulus and thermal properties	[137]

## Data Availability

Not applicable.
